# Noise-Based Image Harmonization Significantly Increases Repeatability and Reproducibility of Radiomics Features in PET Images: A Phantom Study

**DOI:** 10.3390/tomography8020091

**Published:** 2022-04-13

**Authors:** Harald Keller, Tina Shek, Brandon Driscoll, Yiwen Xu, Brian Nghiem, Sadek Nehmeh, Milan Grkovski, Charles Ross Schmidtlein, Mikalai Budzevich, Yoganand Balagurunathan, John J. Sunderland, Reinhard R. Beichel, Carlos Uribe, Ting-Yim Lee, Fiona Li, David A. Jaffray, Ivan Yeung

**Affiliations:** 1 Department of Radiation Oncology, University of Toronto, Toronto, ON M5T 1P5, Canada; yiwen.xu@rmp.uhn.ca (Y.X.); dajaffray@mdanderson.org (D.A.J.); ivan.yeung@rmp.uhn.ca (I.Y.); 2 Techna Institute, University Health Network, Toronto, ON M5G 2C4, Canada; tina.shek@rmp.uhn.ca (T.S.); brandon.driscoll@rmp.uhn.ca (B.D.); brian.nghiem@mail.utoronto.ca (B.N.); 3 Department of Radiology, Weill Cornell Medical College, New York, NY 10021, USA; san2028@med.cornell.edu; 4 Department of Medical Physics, Memorial Sloan Kettering Cancer Center, New York, NY 10065, USA; grkovskm@mskcc.org (M.G.); schmidtr@mskcc.org (C.R.S.); 5 Department of Cancer Physiology, H.L. Moffitt Cancer Center, Tampa, FL 33612, USA; mikalai.budzevich@moffitt.org (M.B.); yoganand.balagurunathan@moffitt.org (Y.B.); 6 Department of Radiology, The University of Iowa, Iowa City, IA 52242, USA; john-sunderland@uiowa.edu; 7 Department of Electrical and Computer Engineering, The University of Iowa, Iowa City, IA 52242, USA; reinhard-beichel@uiowa.edu; 8 Functional Imaging, BC Cancer Agency, Vancouver, BC V5Z 4E6, Canada; carlos.uribe@bccancer.bc.ca; 9 Department of Radiology, The University of British Columbia, Vancouver, BC V5Z 1M9, Canada; 10 Lawson Health Research Institute, London, ON N6A 4V2, Canada; tlee@robarts.ca; 11 Department of Medical Biophysics, University of Western Ontario, London, ON N6A 5C1, Canada; fli222@uwo.ca

**Keywords:** PET radiomics features, feature agreement, image harmonization, repeatability, reproducibility

## Abstract

For multicenter clinical studies, characterizing the robustness of image-derived radiomics features is essential. Features calculated on PET images have been shown to be very sensitive to image noise. The purpose of this work was to investigate the efficacy of a relatively simple harmonization strategy on feature robustness and agreement. A purpose-built texture pattern phantom was scanned on 10 different PET scanners in 7 institutions with various different image acquisition and reconstruction protocols. An image harmonization technique based on equalizing a contrast-to-noise ratio was employed to generate a “harmonized” alongside a “standard” dataset for a reproducibility study. In addition, a repeatability study was performed with images from a single PET scanner of variable image noise, varying the binning time of the reconstruction. Feature agreement was measured using the intraclass correlation coefficient (ICC). In the repeatability study, 81/93 features had a lower ICC on the images with the highest image noise as compared to the images with the lowest image noise. Using the harmonized dataset significantly improved the feature agreement for five of the six investigated feature classes over the standard dataset. For three feature classes, high feature agreement corresponded with higher sensitivity to the different patterns, suggesting a way to select suitable features for predictive models.

## 1. Introduction

Positron emission tomography (PET), unlike other clinical imaging techniques that focus on structural content, reflects the physiological activity of in vivo systems. Extracting imaging biomarkers from PET images through radiomics has become a very active field of research. The motivation behind radiomics is to find “signatures” in an image that could serve as prognostic or predictive biomarkers and therefore augment computer-aided diagnosis, cancer staging, treatment response assessment, and clinical outcomes’ prediction [1].

However, many of the surveyed studies were conducted in a single institution setting, not least because of numerous legal and ethical hurdles to sharing data. Multicenter studies are essential to demonstrate the clinical potential of radiomics, in particular as a predictive tool, as they allow the use of a dataset from another institution to externally validate a prediction model [2,3].

Straightforward pooling of images from different institutions is difficult due to variation in scanner characteristics, differences in acquisition and reconstruction parameters, as well as injected tracer activity between different centers and even within the same centers [2]. This contributes to the fact that, to date, no clear PET radiomic signature has emerged that could predict a clinical outcome in a way that results in a change in clinical decision making. 

For multicenter clinical studies, characterizing the robustness (“agreement”) of radiomics features is essential. The variability of a feature for a given imaging subject or phantom is influenced by all steps in the radiomics workflow from image acquisition and image reconstruction to region of interest segmentation and feature calculation (including various pre-processing steps) [3]. Feature agreement can be quantified using reproducibility and repeatability studies [4]. In PET imaging, most of the literature describes *reproducibility* as the robustness of a feature in images acquired on different PET scanner hardware (often also using different patient populations), different image reconstruction and/or acquisition settings, and potentially different image post-processing methods. In a *repeatability* study (test–retest study), the robustness of features is quantified using repeat images of the same subject (phantom) under identical measurement conditions (same PET scanner, same reconstruction algorithm), but sometimes using different post-processing techniques (including tumor segmentation) before feature calculation. 

To date, at least four review papers have surveyed the literature for reproducibility and repeatability studies. Reference [5] surveyed 41 studies, 18 of which involved PET imaging. Only one PET phantom study was included in the review [6], reference [3] surveyed 42 PET imaging studies and used 21 for a meta-analysis, and reference [7] reviewed 13 studies, 2 of which were clinical PET imaging studies. More recently, PET phantoms with inhomogeneous uptake compartments were used to study radiomics feature agreement across various parameter settings [8,9,10,11]. Most of these studies are combinations of reproducibility and repeatability studies involving some or all of the sources of feature variability associated with the radiomics workflow. 

One important source of feature variability can be attributed to PET image noise, which is inversely proportional to the square root of the scan duration [11]. Variation in scan time had a non-negligible influence on feature agreement, although the level of evidence was considered weak [3]. The reason was the fact that the surveyed literature reached somewhat contradicting conclusions. Reference [9] reported that feature variability increased significantly in low uptake images (high noise) compared to high-uptake (low noise) images; however, filtering the images with a Gaussian in order to reduce image noise does not always result in increased reproducibility of radiomics features, in particular for smaller regions of interest. In addition, in a series of simulations on both CT and PET images of lung cancer patients, reference [12] showed that gray level size zone matrix (GLSZM) features were the most sensitive to uncorrelated noise and that feature variability generally increased with noise level for shape, intensity, and gray level co-occurrence matrix (GLCM) features in both the CT and PET images. On the other hand, for other features, this seemed not always to be the case, at least in images from a single scanner [13]. 

It is generally accepted that harmonization is the key to increasing feature agreement across the data, which in turn is crucially important for the validation of predictive radiomics models. Two main approaches to data harmonization have been described in the literature: harmonization in the image domain (prior to feature extraction) or in the features’ domain, which are described in detail in the review articles [14,15]. The hypothesis is that image harmonization increases repeatability and reproducibility of radiomics features. The first comprehensive harmonization guidelines for PET scanners [16] aimed at standardizing the entire chain of patient preparation, image acquisition, and image reconstruction and were very successful in ensuring good reproducibility of classical imaging biomarkers such as SUVmin and SUVmax [3]. Another method of harmonization is post-processing after image reconstruction, not least because raw list-mode or projection data may no longer be available for reconstruction. Point spread function and time of flight reconstructions are typically used to increase tumor detection, but this can alter SUV values, necessitating a post-processing image filtering technique [17]. However, the authors of [9] found that for the smaller spheres of the NEMA phantom with low uptake (high image noise), many features remained unrepeatable even after post-reconstruction smoothing with a Gaussian filter, hinting at the fact that this approach may be insufficient to fully harmonize radiomics features. Therefore, they concluded that efforts should focus on noise reduction, sometimes even at the cost of spatial resolution, to increase repeatability of radiomics features.

In this work, we hypothesize that mitigating the differences in PET image noise from different scanners is a very efficient harmonization strategy to increase feature agreement. The purpose of this work was therefore three-fold: (1) To generate a rich dataset of PET images of a sophisticated texture phantom, acquired with various image acquisitions protocols, that serves as the basis for image quantification and harmonization studies. (2) To derive radiomics features from the PET phantom images and study their repeatability (across a single scanner) and reproducibility (across different scanners). (3) To explore the effectiveness of a simple image noise harmonization methodology on radiomics feature agreement.

## 2. Materials and Methods

This work was initiated by the PET/CT Subgroup within the Quantitative Imaging Network (QIN). It is devoted to studying bias and variance in quantitative PET imaging through development of imaging phantoms, as well as investigating robust algorithms to extract measurable information from these images [18]. 

### 2.1. Texture Pattern Phantom

Inspired by the uptake of a hypoxia tracer in a tumor, which is generally dependent on perfusion, a phantom was developed to simulate various levels and patterns of uptake. This was achieved by constructing several compartments with various levels of tracer “blockage” in a defined pattern. The compartments were made by threading a large number of 2 mm diameter acrylic rods through a bottom and a top plastic plate. The F-18 tracer would then only occupy the space around the acrylic rods, in effect creating different uptake patterns (“textures”). Four different compartments (“Pattern1”, “Pattern2”, “Pattern3”, and “Pattern4”) were manufactured with approximate fill densities of 28%, 25%, 29%, and 40%, respectively. This approach seemed simpler, although somewhat less flexible, than the approach detailed in [19]. 

**Figure 1:**
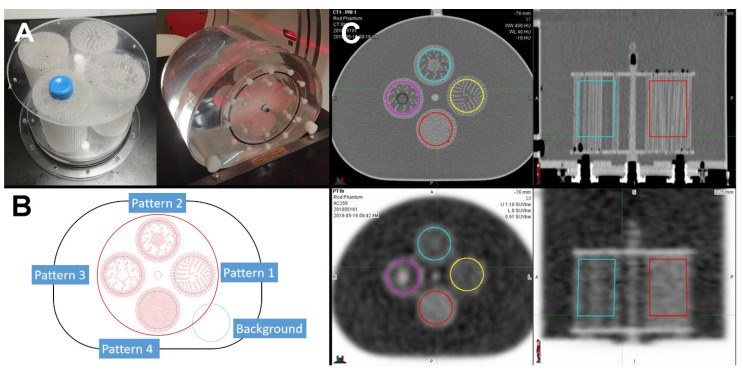
Texture phantom used for this study. (**A**) Photographs of the manufactured cylindrical texture compartments inserted into a standard NEMA phantom shell. (**B**) Each compartment mimics a different distribution of radiotracer uptake due to a different structural pattern of blocked space. (**C**) Axial and coronal CT and PET images of the phantom.

The compartments were inserted into a standard torso NEMA image quality phantom, see Figure 1. Each region resulted in a patterned ROI (region of interest) for radiomic analysis. An identically-shaped region of background was used as an additional fifth ROI.

### 2.2. PET/CT Scans

The phantom was scanned on 10 different PET scanners (9 PET/CT, 1 PET/MRI) in 7 centers across Canada (3) and the United States (4). The scanner models are detailed in Table 1. Each centre contributed to this imaging challenge as part of the QIN (Quantitative Imaging Network) PET/CT Working Group. 

The phantom was transported by mail to the different sites, assembled, and scanned with the guidance of a written manual and PET worksheet. Each participating site scanned the phantom according to several protocols: (a) a “standard” protocol using the institution’s clinical whole body PET imaging protocol with a standard single reconstruction, and (b) several additional PET imaging protocols (using various recommended scanning parameters) resulting in 3 to 4 additional reconstructions per center. A total of 68 PET/CT datasets were submitted to QIPCM at UHN for further analysis. Table 1 shows the scanner make and models of the participating institutions.

### 2.3. Contouring

The four pattern compartments (Patterns 1 to 4) were contoured as cylindrical volumes on one single CT dataset (from a single institution). A background compartment similar in shape was also contoured. These contours were contracted by 5 mm to minimize the partial volume effect. This CT image was rigidly co-registered to the CT of each PET/CT dataset individually, and the contours propagated onto each PET image dataset using MIM Maestro 6.8 (Akron, OH, USA) (Figure 1, panel C). A similar procedure was performed for the PET/MR dataset of institution 9, where the contoured CT was manually registered to the MRI image dataset before contour propagation.

### 2.4. Standard and Harmonized Datasets (for Reproducibility)

The images of each institution’s clinical whole body protocol were used as the *standard dataset* for the radiomics analysis, a total of 10 PET images. The contrast-to-noise ratio (CNR) was calculated across all 68 reconstructions (standard and additional) using the difference in mean activity of the 40% filled region (Pattern-ROI-4; the reference) and the background compartment (Pattern-ROI-5), divided by the standard deviation of the voxel values in the background compartment

(1) $$\mathrm{CNR}=\left|\frac{\mu \left(\mathrm{ROI}\text{-}4\right)-\mu \left(\mathrm{ROI}\text{-}5\right)}{\sigma \left(\mathrm{ROI}\text{-}5\right)}\right|$$

where *μ* and *σ* are the mean and the standard deviation of the PET image values, respectively. An overall mean CNR was also calculated as the average CNR across all 68 reconstructed PET images of all institutions. From each scanner, the reconstruction that deviated the least from the overall CNR mean was selected for the *harmonized dataset* for the radiomics analysis (see Table 1 and Table 2, and Tables S1 and S2 in the Supplementary Materials for more details on the reconstruction algorithms). In order to study feature agreement for two different degrees of harmonization, a subset of the harmonized dataset from 6/10 scanners with smaller CNR variation was selected.

### 2.5. Repeatability Dataset 

On one PET/CT scanner (scanner 7), the phantom was scanned four times at near identical activity concentration levels through periodic top-ups to replenish decayed activity. The scans were then reconstructed from the list-mode data with 2 iterations, 32 subsets, and a Zfilter of 6.4 mm width. Slice thickness was 3.27 mm, the FOV 50 cm, and the matrix size 256, resulting in a pixel size of 1.95 mm. Three different binning times of 10 min, 5 min, and 2 min were used, resulting in three different values for CNR (and therefore mimicking three different noise levels). This resulted in a repeatability dataset of total 3 × 4 = 12 PET images (See Table 3 and Table S3 in the Supplementary Materials for more details on the reconstruction algorithms). 

### 2.6. Radiomics Analysis

For this work, the radiomics features were only computed on the PET images. A total of 107 PET radiomics features were computed using open-source PyRadiomics (2.2.0) [20] from each reconstructed PET image in SUV units using identical PyRadiomics settings. The SUV values were obtained by converting voxel values (Bq/mL) using decay-correction and uptake normalization by a nominal body weight of 50 kg and the injected tracer dose. This resulted in an SUV range across all the images of 0.85–10.15 SUV (min–max) mimicking that in clinical PET images. The 107 features included 14 shape, 18 first order, 24 Gray Level Co-occurrence Matrix (GLCM), 14 Gray Level Dependence Matrix (GLDM), 16 Gray Level Run Length Matrix (GLRLM), 16 Gray Level Size Zone Matrix (GLSZM), and 5 Neighboring Gray Tone Difference Matrix (NGTDM) features. The applied PyRadiomics settings were encapsulated in a parameter YAML file and contained the following: feature extraction from only the original image type, no image normalization before resampling, fixed bin widths of 0.3125 SUV for gray level discretization and histograms, image and mask resampling using default interpolators “sitkBSpline” and “sitkNearestNeighbor”, respectively, and resampled isotropic pixel spacings of 3.5 × 3.5 × 3.5 mm3. As per PyRadiomics documentation, most features are in compliance with the definitions outlined by the Imaging Biomarker Standardization Initiative (IBSI) unless otherwise stated [21,22]. The calculated radiomics features were converted to JSON files (compressed in ZIP format) and MATLAB 2020a (Natick, MA, USA) was then used for quantitative and statistical analysis. Shape features were omitted from the analysis as the compartment shapes were invariant across all PET images. All results are therefore reported for 107 − 14 = 93 features.

### 2.7. Reproducibility and Repeatability Metrics

Inter-scanner feature agreement in the standard and harmonized datasets was assessed using the two-way random, single measures, absolute agreement intraclass correlation coefficient (Shrout and Fleiss ICC(2,1), [23]). The role of the ”judges” in [23] is assumed by the different PET/CT scanners, and the role of the “targets” is assumed by the 5 different pattern-ROIs. ICC(2,1) is a measure of feature value agreement among different ROIs and scanners and expresses essentially a correlation between measurements on the different scanners. In addition, the paired, two-sided Wilcoxon signed-rank test was conducted to test for the null hypothesis that the difference between the ICC(2,1) values between the standard and the harmonized datasets comes from a distribution with zero median. 

In contrast, intra-scanner feature agreement in the repeatability dataset was assessed using the one-way random intraclass correlation coefficient (Shrout and Fleiss ICC(1,1) with k = 4 repeated scan “judges” for the 5 pattern-ROI “targets”). The intraclass correlation coefficient ICC(1,1) is often used to assess feature value absolute agreement between different repeated measurements by the same scanner. Similar to the reproducibility study, the Wilcoxon signed-rank test was also applied for the repeatability assessment to test for statistically significant differences in ICC(1,1) between the 10 min and 2 min PET images.

### 2.8. Pattern Sensitivity

Feature sensitivity to different texture patterns (and independence of volume over which they are calculated) is an essential property of a feature. It was assessed by first calculating the standard deviation *σ* of the values of a radiomics feature *i*, $f_{i}^{j}\left(R\right)$, over the 5 pattern-ROIs *R* in a PET image *j* as follows

(2) $$\sigma_{i}{}^{j}=\sigma^{R}\left[f_{i}^{j}\left(R\right)\right]$$

The mean value of $\sigma_{i}^{j}$ over the 10 images of the standard dataset (S10) and the harmonized dataset (H10) was used as the pattern sensitivity metric $\sigma_{i}^{S}$ and $\sigma_{i}^{H}$, respectively

(3) $$\sigma_{i}{}^{S}=\mu^{j\left\{S10\right\}}\left(\sigma_{i}{}^{j}\right)$$

(4) $$\sigma_{i}{}^{H}=\mu^{j\left\{H10\right\}}\left(\sigma_{i}{}^{j}\right)$$

According to the definition of the intraclass correlation coefficients, higher values of the ICC should correspond to higher values of this pattern sensitivity metric. The Spearman rank correlation coefficient provides a measure of the strength of a monotonic association between ICC(2,1) and the inter-ROI standard deviations $\sigma_{i}{}^{S}$ and $\sigma_{i}{}^{H}$.

## 3. Results

### 3.1. Qualitative Comparison

**Figure 2:**
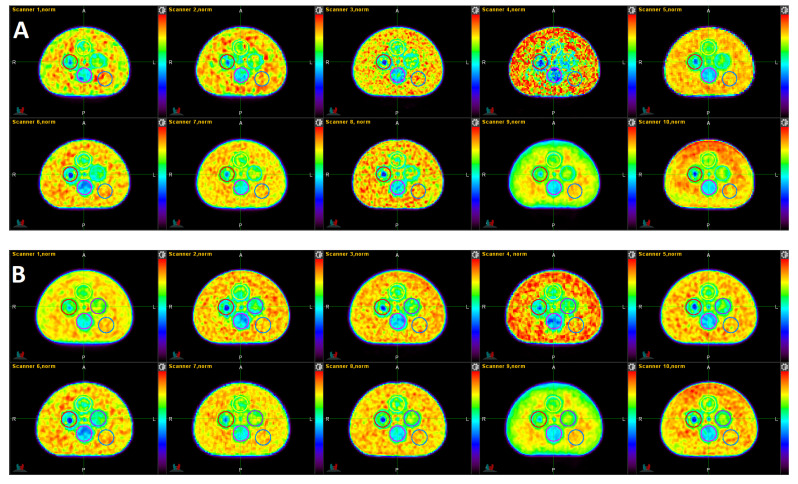
Single slices of the standard (**A**) and harmonized (**B**) datasets. The order of the images follows the order in Table 1 (scanner 1 to 10 from top left to bottom right). The images were normalized by the mean activity of their respective background (Pattern-ROI-5). The displayed color scale is identical for all images and ranges from 0 (black) to 1.22 (red).

A visual qualitative comparison of the 10 PET images in the standard and the 10 PET images in the harmonized datasets are depicted in Figure 2. Scanners 1, 4, 7, and 9 are non-TOF scanners. The CNR, according to Equation (1), of the 10 PET images in panel B are much more similar to each other (7.29 +/− 0.52) than for the 10 PET images in panel A (6.25 +/− 2.23). Image 4 in panel A had the lowest CNR of the set (see Table 2), clearly visible by the conspicuous speckled background. The harmonized version of this image shows a somewhat less noisy background. Image 5 shows the largest CNR of the standard set (CNR = 10.11). Image 9 looks very smooth in both the standard and harmonized sets as it had the longest acquisition time of all scans (10 min). All TOF acquisitions (scanners 2, 3, 5, 6, 8, 10) show the cold center inside Pattern-ROI 3 clearer than the non-TOF scanners. 

### 3.2. Repeatability Analysis

**Figure 3:**
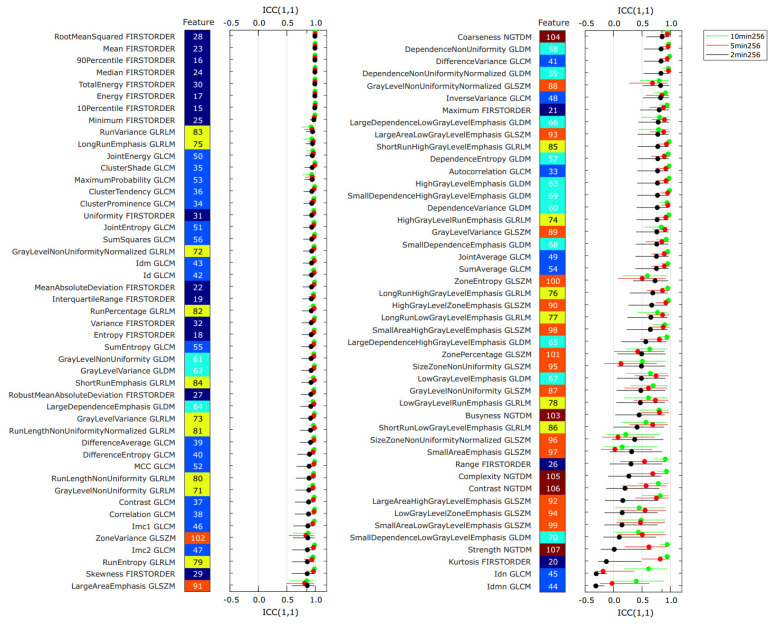
Intraclass coefficient ICC(1,1) for all radiomics features from the PET images in the repeatability dataset. For each feature, three values of ICC(1,1) are plotted: binning time 10 min (green), 5 min (red), and 2 min (black). Error bars indicate the 95% confidence intervals of the ICC values. The results are sorted in descending value of ICC(1,1) for the 2 min binning time (black circles). The feature class of each feature is indicated by the color of the tiles (dark blue: first order, light blue: GLCM, cyan: GLDM, yellow: GLRLM, orange: GLSZM, brown: NGTDM).

Figure 3 shows the intraclass coefficient ICC(1,1) for all features derived from the 12 PET images listed in Table 3 as a ranked plot. For each feature, three ICC values are shown, one for each bin-time. The features are ranked according to the ICC value derived from the 2 min binning time images. 

The mean ICC(1,1) values, averaged over the 107 − 14 = 93 features, were 0.88 ± 0.18, 0.84 ± 0.24, and 0.73 ± 0.30, for the 10 min, 5 min, and the 2 min binning times, respectively. Features derived from the 2 min PET images (noisiest) generally resulted in the smallest ICC among the three different noise levels. This was the case for 81/93 features. In total, 70/93 features of the 10 min images exhibited an ICC(1,1) greater than 0.85, which is usually considered “excellent” feature repeatability. For the 10 min PET images, 80% or more of examined features in the first order, GLCM, GLDM, and GLRLM feature classes demonstrated ICC(1,1) > 0.85, while only 60% and 20% of the NGTDM and GLSZM features were above 0.85, respectively. In contrast, a total of only 48 features of the 2 min binning time showed excellent feature repeatability ICC(1,1) > 0.85. Percentages of features with ICC > 0.85 within the feature classes of first order, GLCM, and GLRLM were 83%, 71%, and 63%, respectively, for 2 min binning time. 

Overall, some feature classes were more affected by PET noise than others. The GLSZM (orange), the NGTDM (brown), and the GLDM features (cyan) are generally found in the bottom half, whereas the first order features and the GLCM features show up in the top half of the ranked plot. For 2 min binning time, not more than 20% of the features in each of the feature classes GLDM, GLSZM, and NGTDM were > 0.85. 

The paired Wilcoxon signed-rank test suggested a statistically significant improvement in feature ICC(1,1) value from the 2 min to the 10 min scan. The average ICC(1,1) value improved by 0.15 ± 0.22 (*p* < 10^−10^), see also Table S4 in the Supplementary Materials. The improvement from the 2 min to the 10 min scan is significant for the first order, GLCM, GLDM, and the GLRLM, but not for the GLSZM, and NGTDM feature classes, see Table S5 in the Supplementary Materials. Table S6 in the Supplementary Materials lists the values for ICC(1,1) for all 93 features and all three binning times. 

### 3.3. Reproducibility Analysis

The reproducibility analysis was conducted for a total of four different datasets: for the standard dataset (S10), the harmonized dataset (H10), and then a subset of six scanners each (S6 and H6, see Table 4 for the overview). For H6, a subset of six PET images (and their scanners) was selected from H10 such that the mean CNR of the subset was closest to the mean CNR of the full dataset F. For S6, the six PET images from the same scanners as for H6 were selected from the standard dataset S10. 

**Figure 4:**
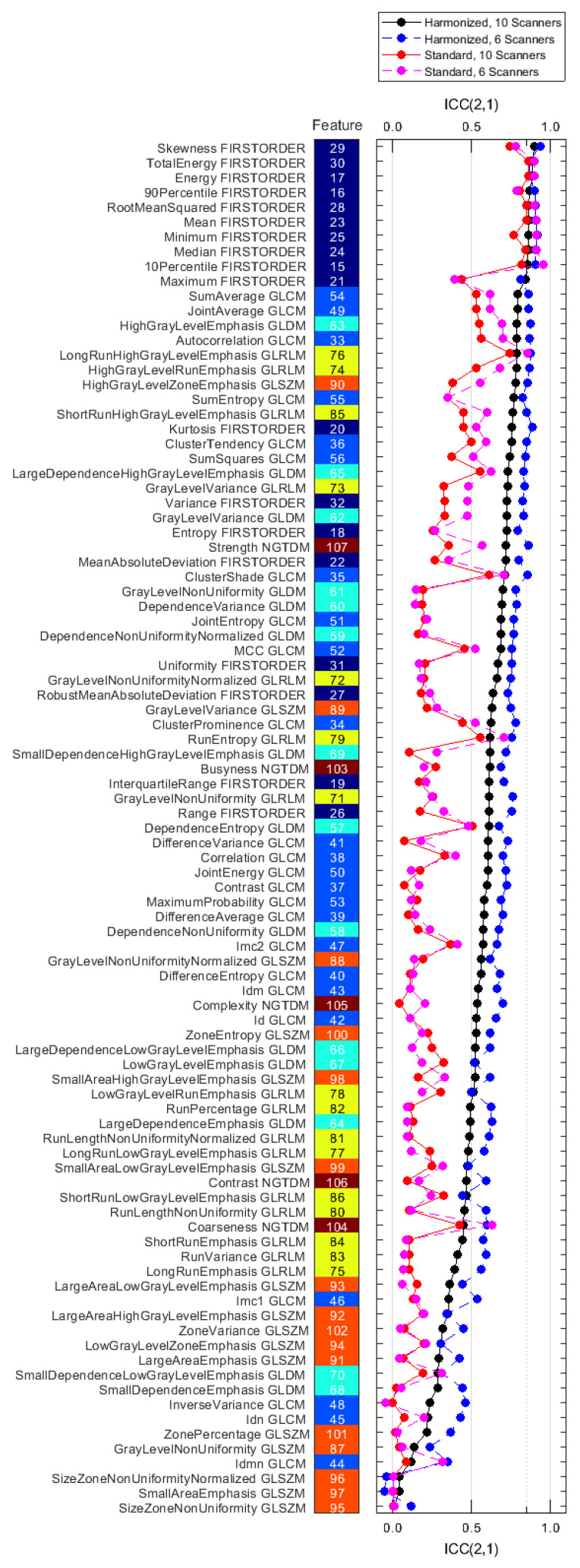
ICC(2,1) values for all features for the reproducibility datasets S10, H10, S6, and H6 (Table 4), ranked by decreasing value of the H10 dataset (solid black). The feature class of each feature is indicated by the color of the tiles (dark blue: first order, light blue: GLCM, cyan: GLDM, yellow: GLRLM, orange: GLSZM, brown: NGTDM).

Figure 4 shows the reproducibility for each computed feature for each of the four datasets. All numerical results are also listed in Table S7 in the Supplementary Materials. 

The mean ICC(2,1) values, averaged over the 93 features, were 0.30 ± 0.24, 0.58 ± 0.21, 0.34 ± 0.27, and 0.67 ± 0.21 for the S10, H10, S6, and H6 datasets, respectively. Only 2/93 features for the S10 and 9/93 features for the H10 dataset had ICC values larger than 0.85. For the S6 and H6 datasets, these values were 8/93 and 19/93 features, respectively. 

**Figure 5:**
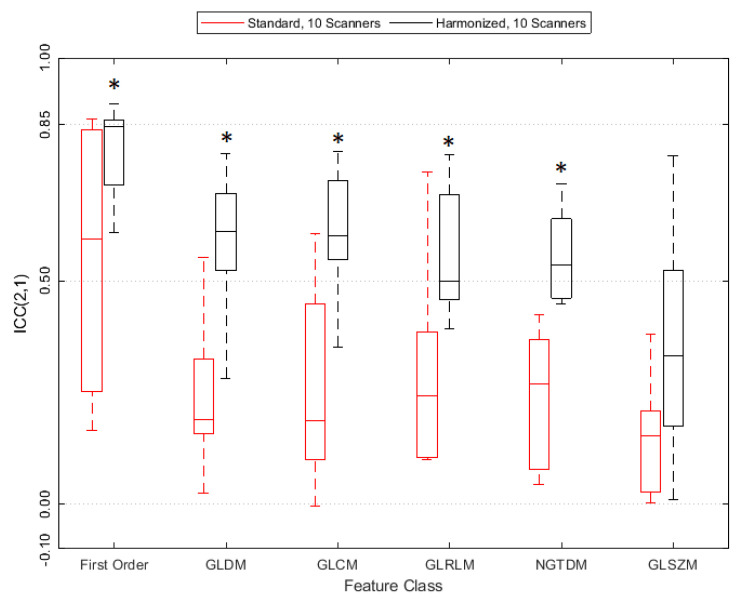
ICC(2,1) values grouped by radiomics feature class. A star (*) indicates statistical significance according to the paired Wilcoxon signed-rank test.

The paired Wilcoxon signed-rank test suggested a statistically significant improvement in ICC(2,1) value from the standard (S10) to the harmonized (H10) dataset, see also Table S8 in the Supplement. The average feature ICC(2,1) value improved by 0.28 ± 0.16 (*p* = 5.57 × 10^−17^). Figure 5 shows that reproducibility improves for all feature classes except GLSZM on a statistically significant level, the detailed results are in Table S9 in the Supplement. Figure 4 also demonstrates that tightening the range of CNR in the datasets (S6 and H6) further improves feature agreement. ICC(2,1) is larger for 85/93 features when using the H6 versus the H10 dataset, and for 64/93 features when using the S6 versus the S10 dataset.

### 3.4. Feature Pattern Sensitivity

**Figure 6:**
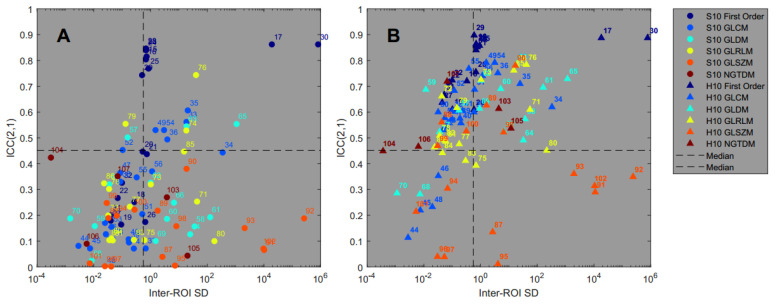
Feature reproducibility across different scanners (ICC(2,1)) versus pattern sensitivity (inter-ROI standard deviation) for all features in the standard (**A**) and harmonized (**B**) datasets.

Figure 6 shows each feature’s ICC(2,1) as a function of the pattern sensitivity in terms of the mean inter-ROI standard deviation according to Equations (3) and (4). All numerical results are also listed in Table S7 in the Supplementary Materials. 

The results of the Spearman rank correlation test showed that the features of the first order (rho = 0.79 and 0.74) and GLCM (rho = 0.67 and 0.84) feature classes show a significant association between ICC(2,1) and pattern sensitivity for both the standard and harmonized datasets. The features of the GLDM class are not correlated for the S10 dataset (Spearman’s rho = 0.25), but are just slightly above the significance level of 0.05 for the harmonized dataset (Spearman’s rho = 0.53). The full list of Spearman’s rank correlation test results can be found in Table S10 in the Supplementary Materials. 

## 4. Discussion

We have studied the feature agreement of radiomics features calculated on PET images by conducting a repeatability and a reproducibility study using a purpose-built pattern phantom. The main purpose of the work was to investigate the feature agreement across non-harmonized (standard) and harmonized datasets. 

### 4.1. Feature Calculation

As shown by [24] in their NEMA phantom study, bin width and pixel/voxel size can significantly influence the variability of a feature across PET images from different scanners, even for a relatively simple phantom with spherical inhomogeneities. 

Radiomics feature calculation in this work was performed for fixed bin widths (in units of an SUV range). Fixed bin widths, as opposed to fixed number of bins, are reported to be more suitable for images from clinical settings due to variable SUV ranges that can occur for variable administered activity concentration and scan time after injection [25], [26] but also for images from a standardized phantom [9]. The chosen fixed bin width of 0.3125 SUV for our analysis enabled us to cover the clinically relevant SUV range of 0–20 in 64 bins. 

Pixel spacing across all PET images in the standard dataset varied between 1.95–5.49 mm and slice thickness between 2.03–5.00 mm. In the harmonized datasets, the pixel spacing range was 1.95–3.91 mm. Following [27], resampling pixel spacing to isotropic voxels further reduces effects arising from using a different number of voxels within the ROIs. 

### 4.2. Harmonization Method

The results of the simple method in Figure 3 and Figure 4 demonstrated clearly that PET image noise and its variation between PET images directly influences the reproducibility and repeatability of a feature. A very simple harmonization method based on minimizing the differences in CNR in the PET images was applied. The statistically significant differences between the standard (clinically preferred) and the harmonized datasets clearly echoes the conclusions in reference [9] for the homogeneous NEMA phantom. In addition, tighter harmonization criteria in terms of CNR range further improve feature agreement.

Our study design allowed for a different selections of images from each institution who provided images with a wide range of CNRs. Therefore, no further post-processing method was necessary to generate the harmonized dataset. Table 5 provides the image acquisition and reconstruction settings for this particular group of PET scanners that result in a “harmonized” CNR in the resulting PET images. These settings could be used if a multi-institutional clinical trial was to be conducted with these institutions. The usual standard reconstruction at each centre can still be used for interpretation by the local physician and the harmonized reconstruction sent to the imaging core lab for analysis. Such a process, therefore, does not have to compromise between clinical and trial requirements. However, using original PET images might not always be practical. Harmonization of the dataset by post-processing images would not require the institutions to acquire multiple images with different settings. Our results show that a particular class of post-processing algorithms, i.e., one that harmonizes noise in PET images, might have the potential to increase feature agreement. It remains to be seen if post-processing techniques such as a convolution with a Gaussian blurring filter can achieve a similar result. This will be pursued in future work, ultimately validating image post-processing methods for harmonization. 

A ComBat-type harmonization method [28] would not be applicable for our datasets and study design. Our work is a phantom study and the number of unique regions of interest per scanner is only one. Hence, the variation in feature values for a particular ROI was caused by the different acquisition and reconstruction techniques and not due to any underlying variability of the ROI itself, as would be the case for clinical images from different patients.

### 4.3. Pattern Sensitivity

Ideally, the pixel values in a PET image reflect the nature of an underlying spatial arrangement of a physiological process, and radiomics features should provide a genuine characterization of this pattern. However, in addition to confounding image noise, it has been demonstrated that by their inherent definition, the values of many radiomics features are a surrogate of the volume over which they are calculated [29,30]. Orlhac et al. [30] demonstrated that several texture features are highly correlated to metabolic volume independent of tumor type; although for larger tumor volumes, metabolic volume and texture become independent factors [31]. A similar conclusion was also reached in [6] where the volume dependency could even demonstrate very different behavior depending on the feature. This suggests that the absolute values of the ICC are subject to the variability arising from different volume sizes, as also shown in [9]. In our study, volume was not a confounding factor, as the different pattern-ROIs over which the features were calculated were identical in volume and shape. This allowed us to investigate the behavior of a feature and feature agreement for different underlying physical patterns alone.

The results from Figure 6 demonstrate that higher feature agreement across different PET scanners as measured by the ICC is associated with higher pattern sensitivity as measured by the mean inter-ROI standard deviation of the feature values. This is, in particular, the case for the harmonized dataset and for the three feature classes First Order, GLCM, and GLDM. Hence, for these features the variability of its values that is introduced by the different texture pattern is much larger than the variability that is introduced by the different reconstruction algorithms and image noise. This is very desirable and provides a selection of features that might be suitable for use in a predictive model. As an example, entropy has sometimes been deemed a good candidate for predicting a clinical event, for example, in reference [32]. The three entropy features “Entropy” (first order), “SumEntropy” (GLCM) and “JointEntropy”, (GLCM), show excellent repeatability with ICC(1,1) larger than 0.93 in our study. The ICC(2,1) improved significantly after harmonization for all three features, as did the pattern sensitivity. 

In general, however, it is very difficult to compare results between studies, even between phantom studies, as feature extraction algorithms and statistical analysis methods are usually different. For example, Gallivanone et al. [8] used an in-house developed MATLAB code and a coefficient-of-variation (COV) analysis to characterize feature agreement between different reconstructions. The GLCM features Entropy and GLCM Homogeneity (Inverse Difference) rank high, whereas in our study (Figure 4) GLCM SumEntropy ranks high among GLCM features but GLCM Inverse Difference ranks low. In addition, the best of the GLRLM features in [8], in terms of COV, were ShortRunEmphasis and LongRunEmphasis. Both of these features scored very low for us in Figure 4 even after harmonization. One possible reason for these discrepancies is that all reconstructions in [8] were generated on the same PET scanner, whereas the PET images in our study were reconstructed on different PET scanners. 

## 5. Conclusions 

PET image noise and its variation between PET images directly influences the reproducibility and repeatability of radiomics features. A simple harmonization method of the (clinical) standard datasets based on minimizing the differences in CNR in the PET images significantly increases feature agreement. 

## Figures and Tables

**Table 1:** GE = GE Medical Systems (Waukesha, WI, USA). Siemens = Siemens Medical Solutions USA, Inc. Malvern, PA, USA.

Scanner	Manufacturer	Manufacturer Model Name	TOF Capability
1	GE	Discovery 600	No
2	GE	Discovery 690	Yes
3	GE	Discovery MI	Yes
4	GE	Discovery STE	No
5	GE	Discovery 710	Yes
6	GE	Discovery MI DR	Yes
7	GE	Discovery 610	No
8	Siemens	Biograph40_mCT	Yes
9	Siemens	Biograph_mMR	No
10	Siemens	Biograph64_mCT	Yes

**Table 2:** Reconstruction parameters and contrast-to-noise ratios for the standard dataset (10 PET images). ScNo = scanner number from Table 1. TOF = time of flight acquisition, ZFilter = post-reconstruction filter, NIt = number of iterations, NSubS = number of subsets, FOV = field of view, M = matrix size, P = pixel spacing (pixel size), S = slice thickness, Time = acquisition time (per bed position), CNR = contrast-to-noise ratio according to Equation (1).

ScNo	TOF	ZFilter (mm)	NIt	NSubS	FOV (cm)	M	P (mm)	S (mm)	Time (min)	CNR
1	No	6.4	2	32	70	192	3.65	3.27	2.5	4.62
2	No	6.4	2	32	70	192	3.65	3.27	2.5	4.50
3	Yes	5	3	16	50	256	1.95	2.79	3	4.38
4	No	4	2	20	50	128	3.91	3.27	5	3.47
5	Yes	6.4	2	16	70	128	5.47	3.27	3	10.11
6	Yes	6.4	2	32	70	192	3.65	3.27	3	7.02
7	No	6.4	2	32	50	256	1.95	3.27	5	6.39
8	Yes	5	3	21	81.5	256	3.18	5	2	4.37
9	No	6	3	21	71.8	256	2.80	2.03	10	8.39
10	Yes	5	2	21	81.5	200	4.07	5	5	9.26
CNR Mean										6.25
CNR Standard Deviation										2.23

**Table 3:** Contrast-to-noise ratios for the repeatability dataset (12 PET images). Time = acquisition time (per bed position), R = reconstruction, CNR = contrast-to-noise ratio according to Equation (1).

Time (min)	CNR					
	R1	R2	R3	R4	Mean	Std Dev
10	7.74	8.09	8.41	8.01	8.06	0.28
5	5.61	5.83	5.80	5.45	5.67	0.18
2	3.86	3.71	3.70	3.53	3.70	0.13

**Table 4:** Summary of datasets for the reproducibility analysis.

Label	Dataset	Scanners	Number PET Images	Average CNR +/− 1 SD
F	Full dataset	1–10	68	7.53 ± 2.38
S10	Standard dataset	1–10	10	6.25 +/− 2.23
H10	Harmonized Dataset	1–10	10 (*)	7.29 +/− 0.52
S6	Subset of S10	1,2,3,5,8,10	6	6.21 ± 2.71
H6	Subset of H10	1,2,3,5,8,10	6 (*)	7.38 ± 0.25

(*) closest to average CNR of F.

**Table 5:** Reconstruction parameters and contrast-to-noise ratios for the harmonized dataset (10 PET images). ScNo = scanner number according to Table 1. TOF = time of flight scanner, ZFilter = post-reconstruction Zfilter, NIt = number of iterations, NSubS = number of subsets, FOV = field of view, M = matrix size, P = pixel spacing (pixel size), S = slice thickness, Time = acquisition time (per bed position), CNR = contrast-to-noise ratio according to Equation (1).

ScNo	TOF	ZFilter (mm)	NIt	NSubS	FoV (cm)	M	P (mm)	S (mm)	Time (min)	CNR
1	No	6.4	3	16	50	256	1.95	3.27	5	7.29
2	Yes	4.6	3	16	50	256	1.95	3.27	5	7.09
3	Yes	6.4	3	16	50	256	1.95	2.79	5	7.29
4	No	6	3	14	50	128	3.91	3.27	10	6.3
5	Yes	6.4	3	16	50	256	1.95	3.27	3	7.64
6	Yes	6.4	2	32	70	192	3.65	3.27	3	7.02
7	No	6.4	2	32	50	128	3.91	3.27	5	6.95
8	Yes	6	3	21	81.5	256	3.18	5	5	7.25
9	No	6	3	21	71.8	256	2.80	2.03	10	8.39
10	Yes	6	3	21	50.9	256	1.99	3	5	7.72
CNR Mean										7.29
CNR Standard Deviation										0.52

## Data Availability

Not applicable.

## Supplementary Materials

The following supporting information can be downloaded at: https://www.mdpi.com/article/10.3390/tomography8020091/s1. Table S1: PET acquisition and reconstruction settings for the standard dataset S10; Table S2: PET acquisition and reconstruction settings for the harmonized dataset H10; Table S3: PET acquisition and reconstruction settings for the repeatability dataset; Table S4: Summary of Wilcoxon signed-rank test statistics; Table S5: Summary of Wilcoxon signed-rank test statistics grouped by feature class; Table S6: ICC(1,1) values for all features; Table S7: ICC(2,1) values for all features; Table S8: Summary of Wilcoxon signed-rank test statistics; Table S9: Summary of Wilcoxon signed-rank test statistics grouped by feature class; Table S10: Results of the Spearman rank correlation test.
