# Supplementary figures and images for: Noise-Based Image Harmonization Significantly Increases Repeatability and Reproducibility of Radiomics Features in PET Images: A Phantom Study
Source: Tomography. 2022 Apr 13;8(2):1113–28. doi: 10.3390/tomography8020091 (PMC9025788; doi:10.3390/tomography8020091)

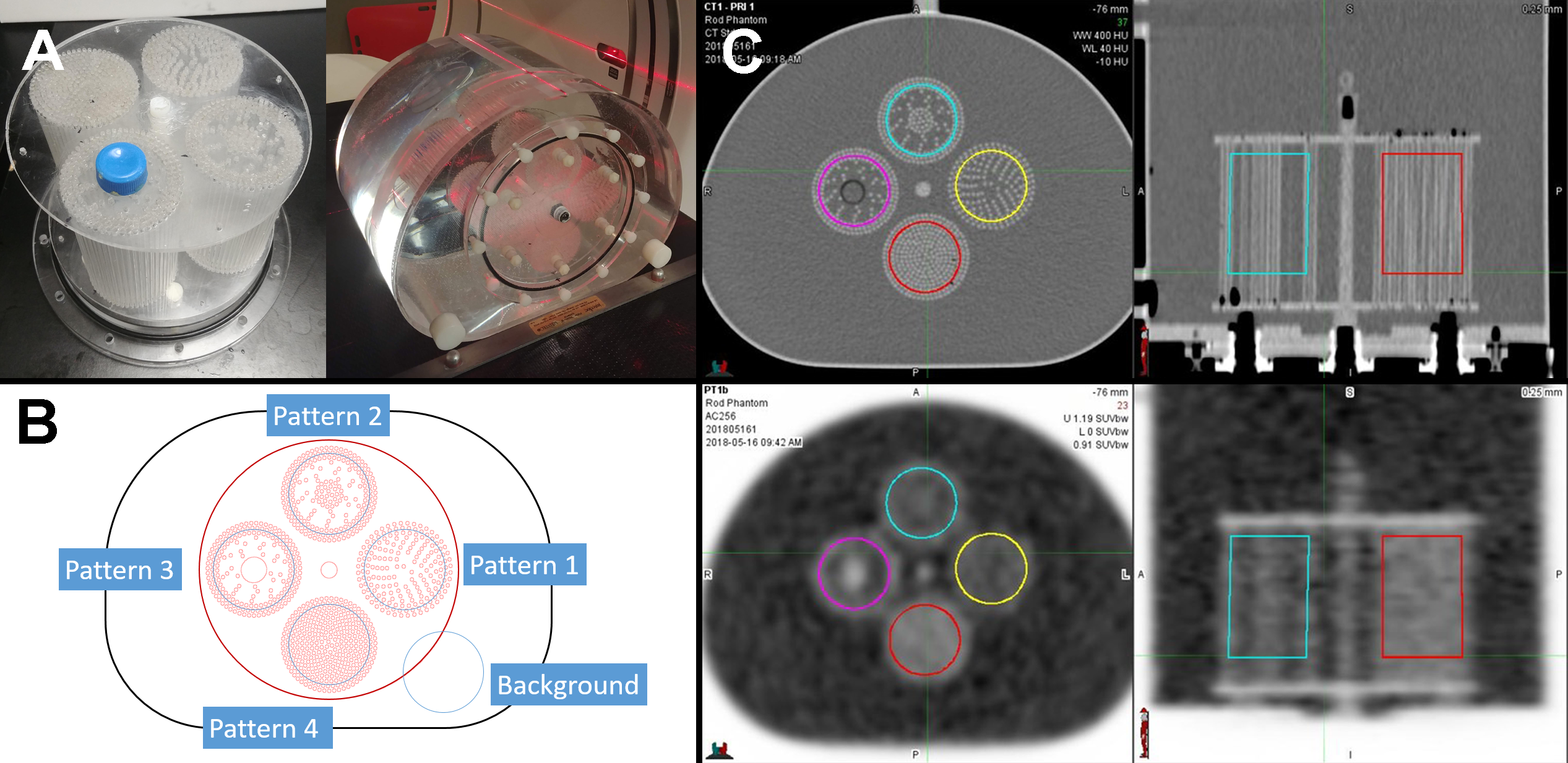

Supplement: Supplementary file 1 [file tomography-08-00091-s001.zip › FigureS1.png]

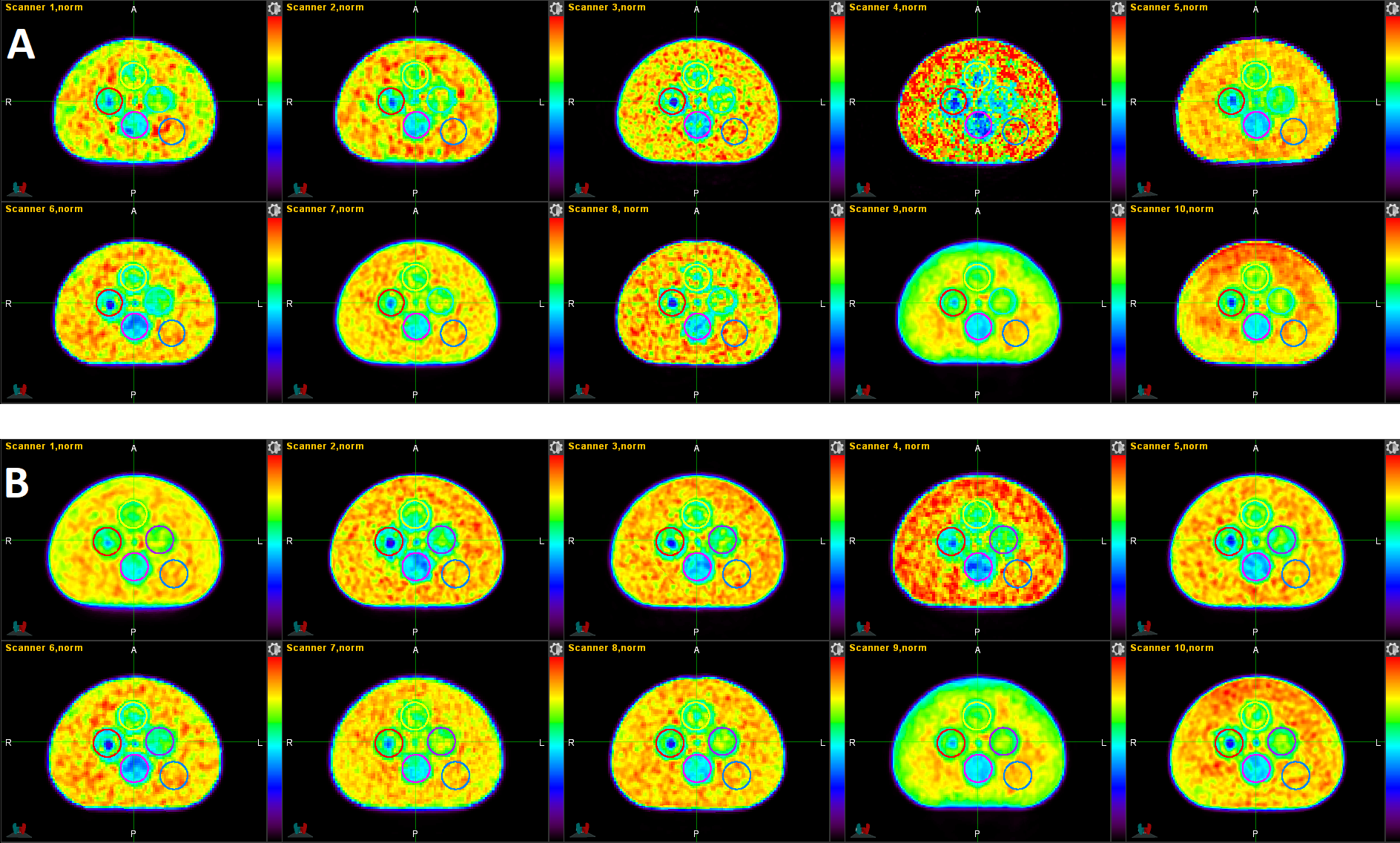

Supplement: Supplementary file 1 [file tomography-08-00091-s001.zip › FigureS2.png]

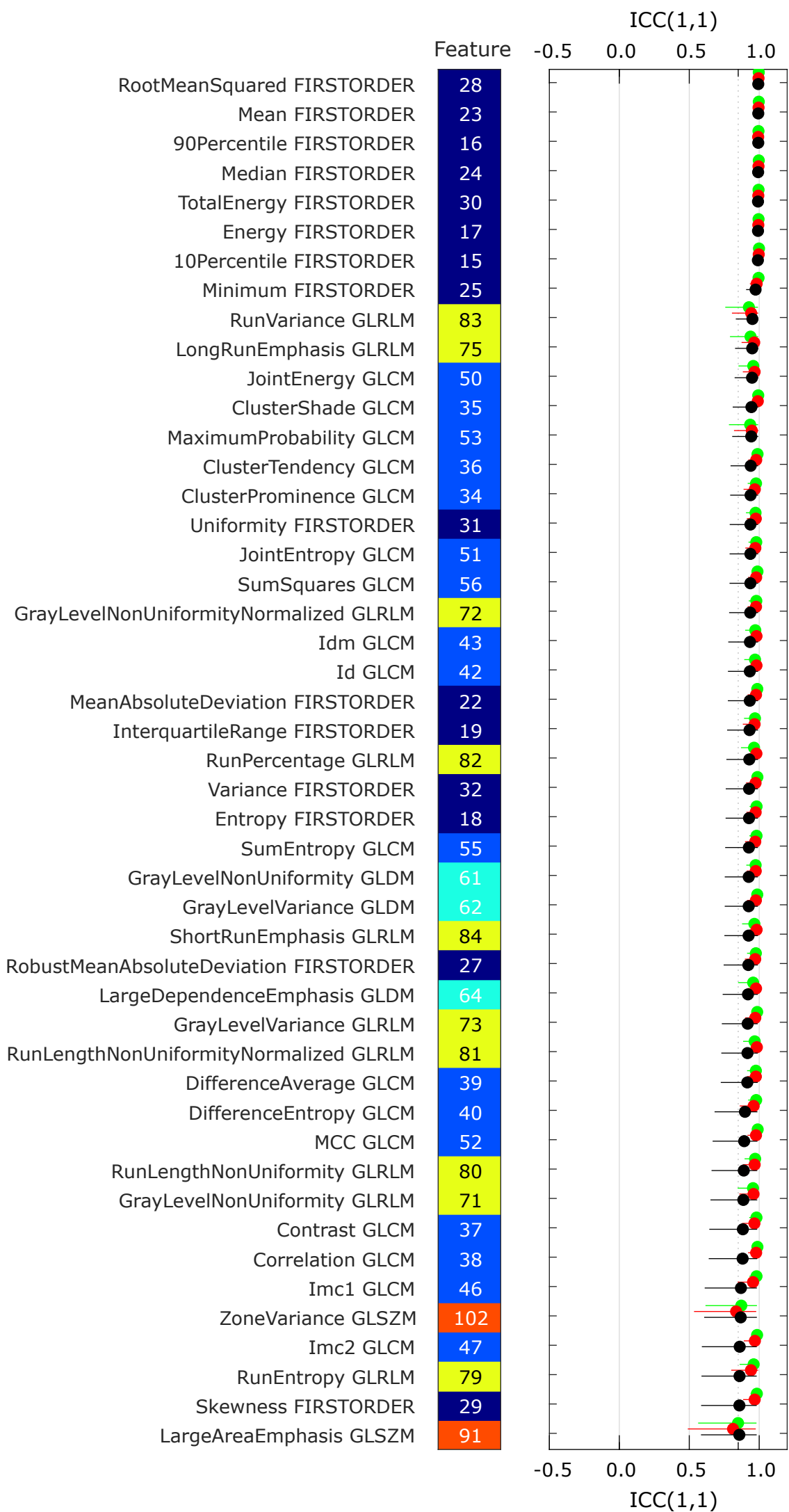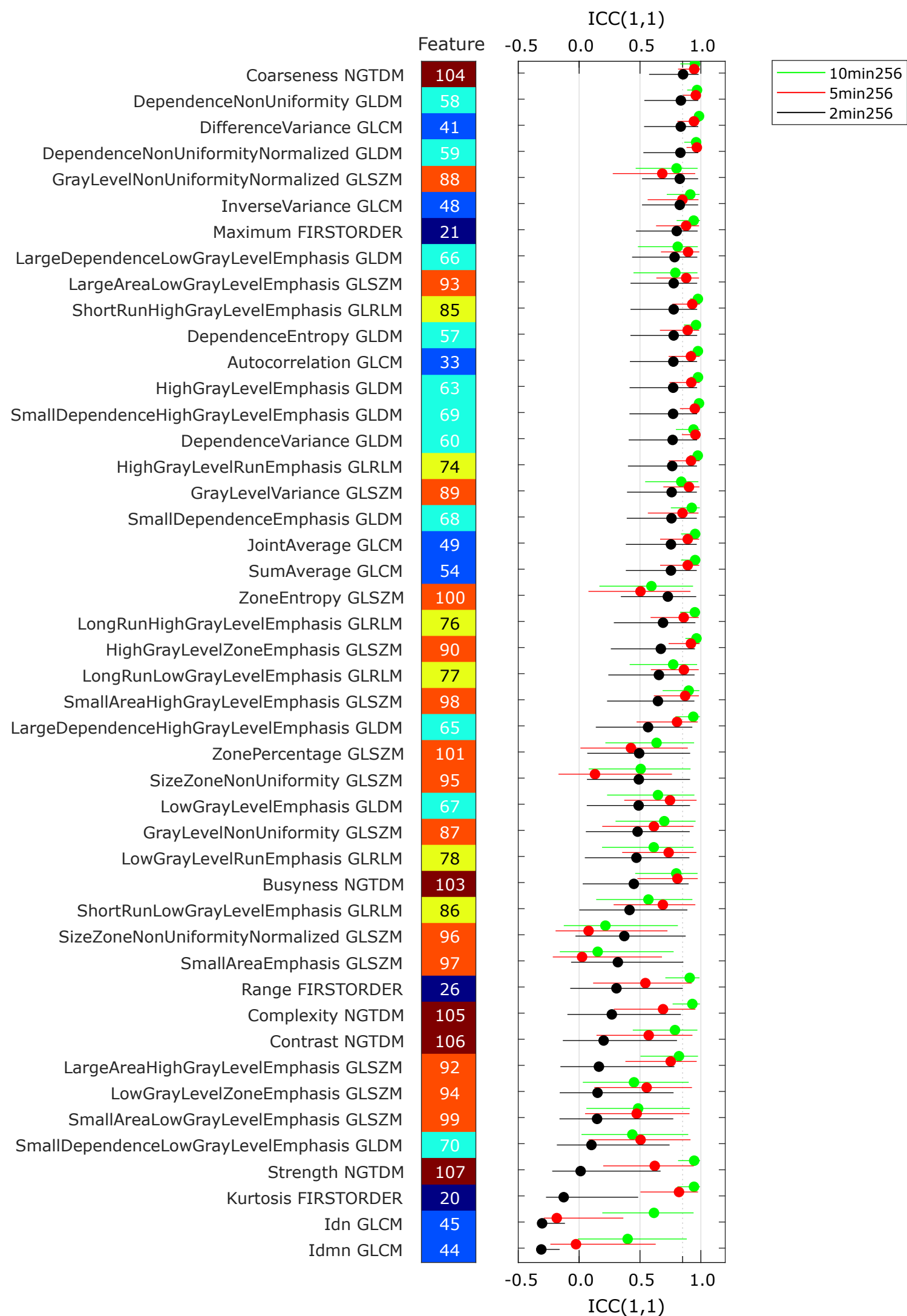

Supplement: Supplementary file 1 [file tomography-08-00091-s001.zip › FigureS3.pdf]

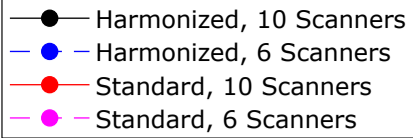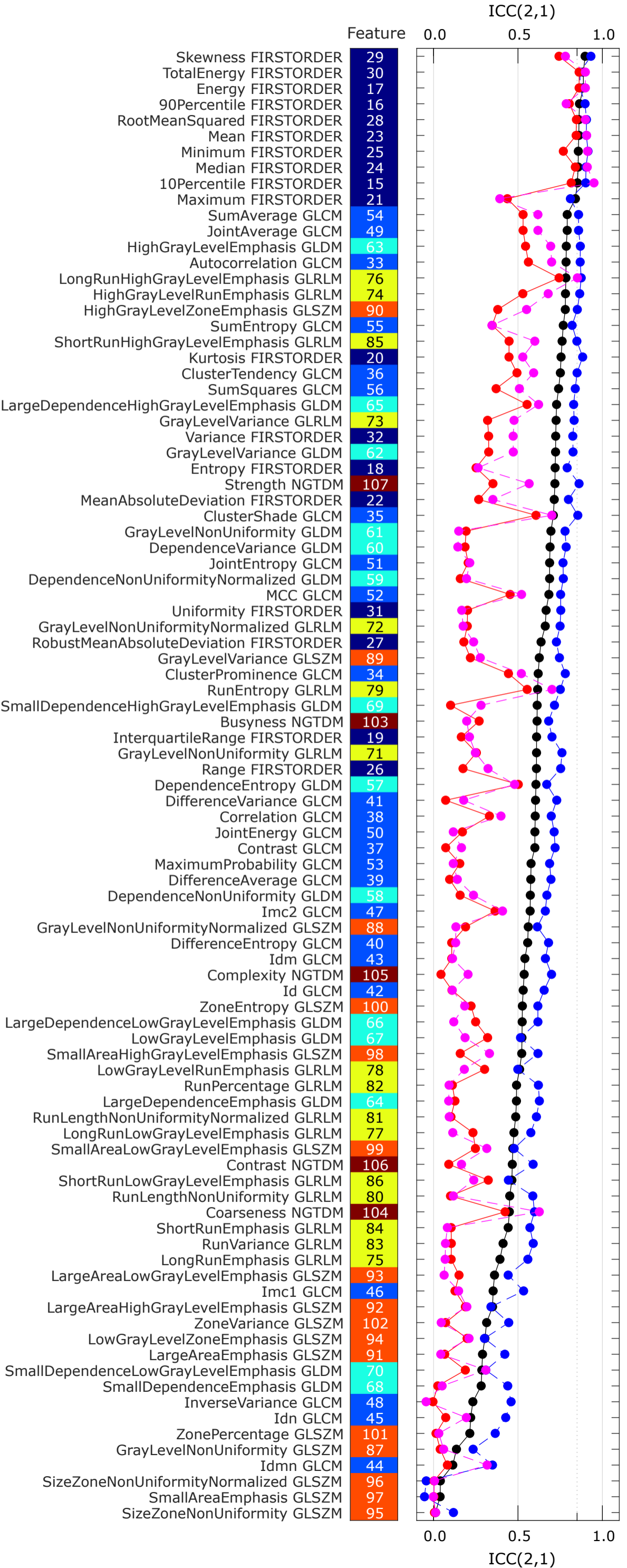

Supplement: Supplementary file 1 [file tomography-08-00091-s001.zip › FigureS4.pdf]

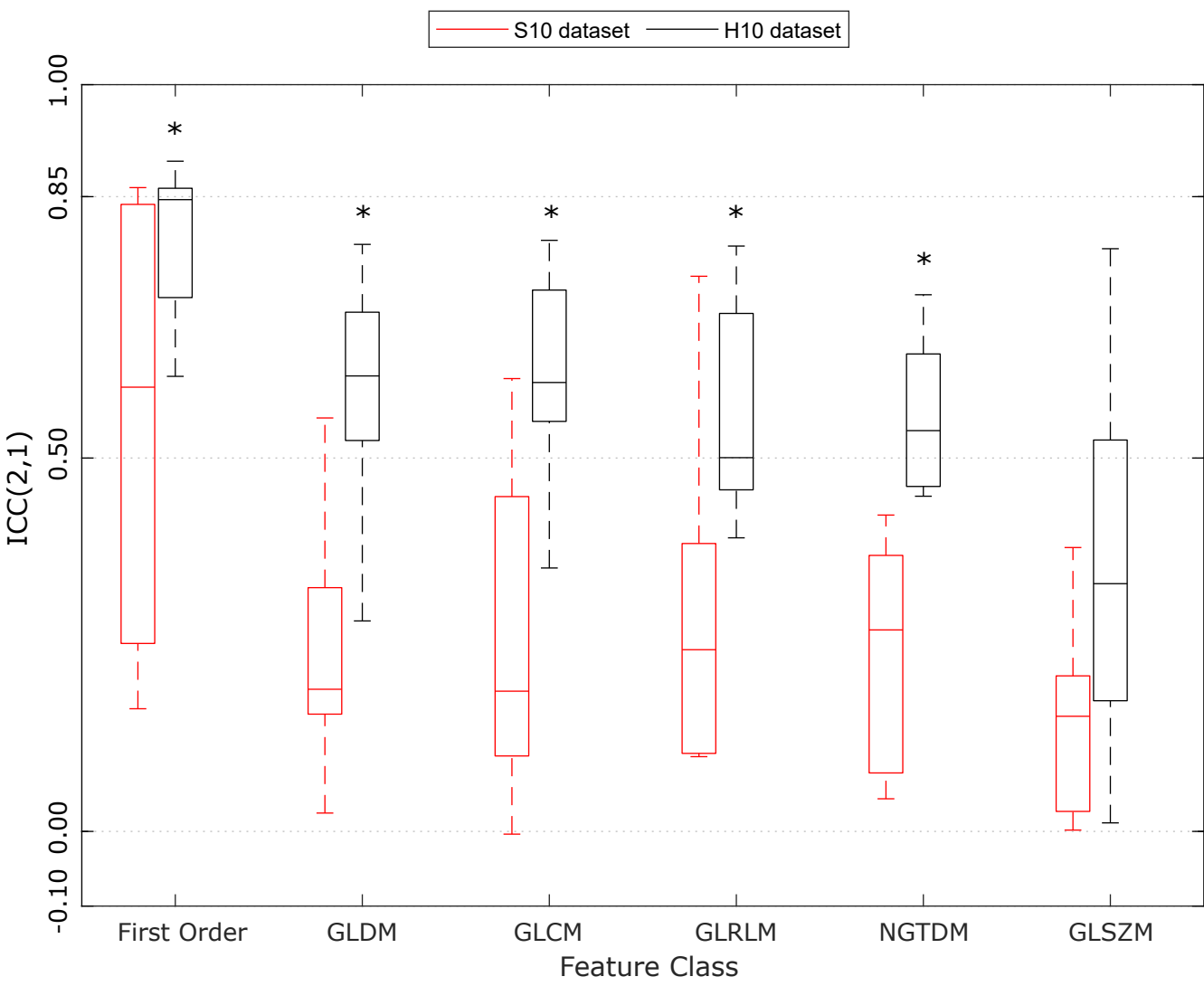

Supplement: Supplementary file 1 [file tomography-08-00091-s001.zip › FigureS5.pdf]

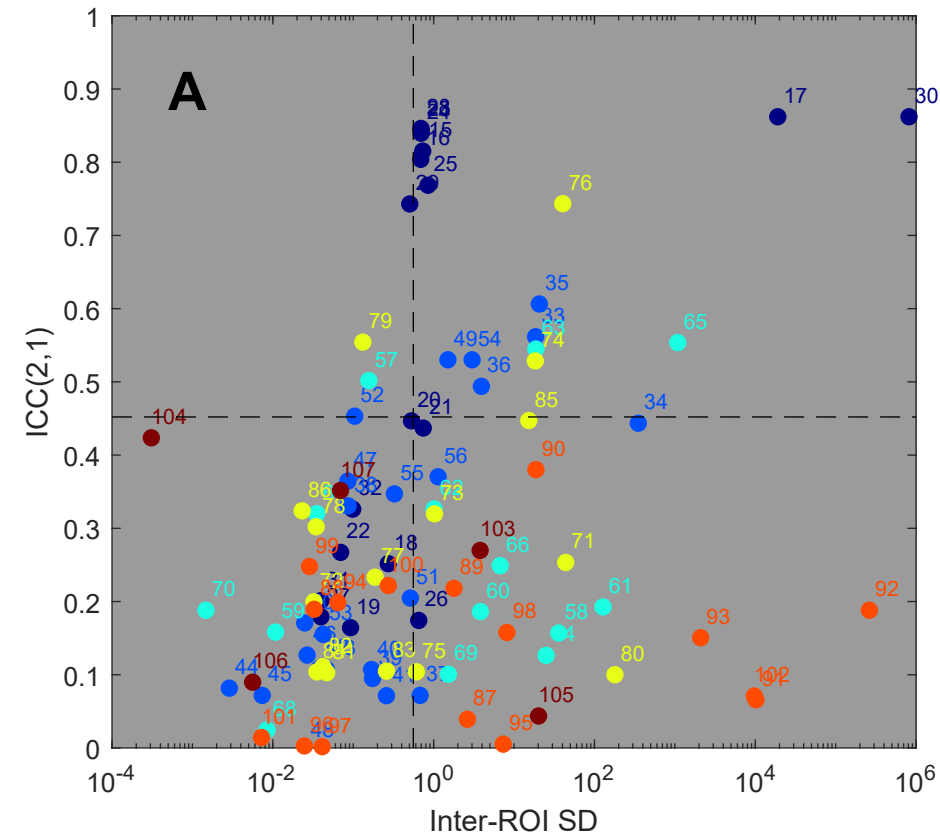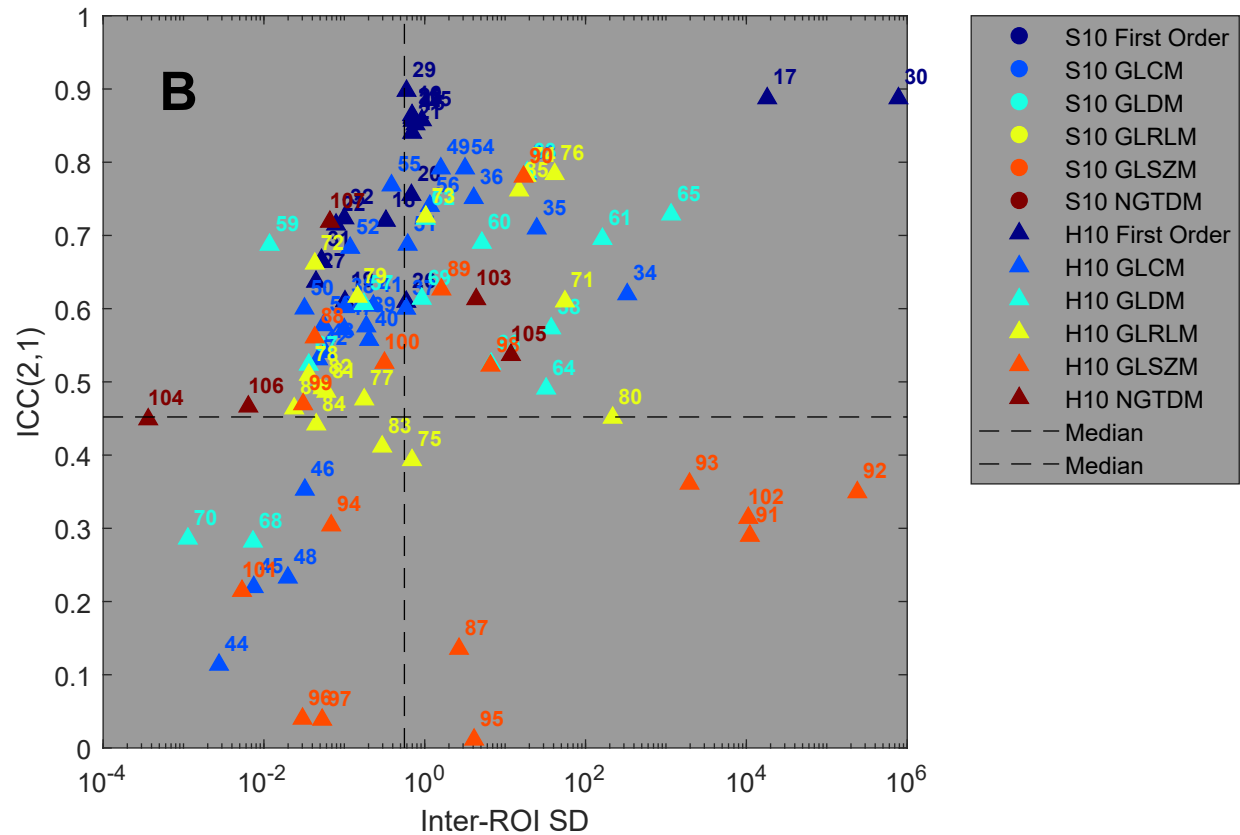

Supplement: Supplementary file 1 [file tomography-08-00091-s001.zip › FigureS6.pdf]
